# Lifting COVID-19-associated non-pharmaceutical interventions: potential impact on notifications of infectious diseases transmitted from person to person in 2022 in Bavaria, Germany

**DOI:** 10.3389/fpubh.2024.1437485

**Published:** 2024-07-31

**Authors:** Judith Hausmann, Achim Dörre, Katharina Katz, Sarah van de Berg

**Affiliations:** ^1^Institute for Medical Information Processing, Biometry, and Epidemiology - IBE, LMU Munich, Munich, Germany; ^2^Pettenkofer School of Public Health, Munich, Germany; ^3^Bavarian Health and Food Safety Authority (LGL), Munich, Germany; ^4^Department of Infectious Disease Epidemiology, Postgraduate Training for Applied Epidemiology (PAE), Robert Koch Institute, Berlin, Germany

**Keywords:** infectious diseases, surveillance, pandemic, non-pharmaceutical interventions, influenza, chickenpox, norovirus gastroenteritis, rotavirus gastroenteritis

## Abstract

**Background:**

The COVID-19 pandemic and associated non-pharmaceutical interventions (NPIs) have led to substantial decreases in case numbers of infectious diseases in several countries worldwide. As NPIs were gradually lifted, intense or out-of-season outbreaks of respiratory and gastrointestinal diseases were reported, raising the hypothesis of a potential catch-up effect of infections. By analysing surveillance data from the federal reporting system for notifiable infectious diseases, we aimed to assess the potential impact of lifting COVID-19 associated NPIs on notifications of selected infectious diseases in Bavaria, 2022.

**Methods:**

We compared influenza, chickenpox, norovirus gastroenteritis, rotavirus gastroenteritis weekly case numbers in a pre-pandemic period (2016–2019) and 2022 using two time series analyses approaches: (i) a predictive model forecasting weekly case numbers for the pandemic years 2020–2022, based on 2016–2019 data, (ii) interrupted time series model, based on 2016–2022 data, including a term per pandemic period.

**Results:**

In 2022, incidence rates were higher compared to pre-pandemic period for influenza (IRR = 3.47, 95%CI: 1.49–7.94) and rotavirus gastroenteritis (IRR = 1.36, 95%CI: 0.95–1.93), though not significant for rotavirus gastroenteritis. Conversely, case numbers remained significantly below pre-pandemic levels for chickenpox (IRR = 0.52, 95%CI: 0.41–0.65) and norovirus gastroenteritis (IRR = 0.59, 95%CI: 0.42–0.82). Seasonality changed notably for influenza, showing an earlier influenza wave compared to pre-pandemic periods.

**Conclusion:**

The lifting of NPIs was associated with heterogenic epidemiological patterns depending on the selected disease. The full impact of NPIs and their discontinuation may only become clear with continued monitoring and assessment of potential additional contributing factors.

## Introduction

During the coronavirus disease 2019 (COVID-19) pandemic the number of notified cases of several infectious diseases with different modes of transmission decreased in 2020–2021 compared to earlier years in Germany and other countries (1–3). This was also seen in the German federal state Bavaria with more than 13 million inhabitants (4). For diseases transmitted from person-to-person, the reduction of notified cases may, to a substantial extent, be explained by decreased transmission due to non-pharmaceutical interventions (NPIs) such as temporary lockdowns, travel bans, contact restrictions, use of personal protective equipment and increased (hand/cough) hygiene but also under-detection due to reduced health care seeking behavior (4).

As countries gradually discontinued NPIs in 2021 and 2022, rebound effects and unusual out-of-season outbreaks of respiratory and gastroenterological infections were first simulated in modeling studies (5, 6) and later documented in several countries worldwide. These dynamics were observed for respiratory syncytial virus (RSV) (7), rhinovirus infections (8), hand, foot, and mouth disease (2), norovirus gastroenteritis (9), and influenza (10). As children were predominantly affected (7–9), concerns arose that this phenomenon would appear in other infectious diseases, like rotavirus gastroenteritis or chickenpox as well (11). While likely a number of factors contributed to the resurgence of several infectious diseases, one explanation is decreased community immunity caused by NPIs during the first two pandemic years, which may have resulted in increased susceptibility to certain diseases (3). In Germany, rhinovirus and RSV infection numbers rose comparably early in 2021, reaching a peak already in the summer months (12). High numbers of hospitalizations in late 2022 and record high work absence resulting from respiratory infections mainly caused by RSV and influenza were reported (12).

In Germany, each federal state may enact NPIs in addition to national measures. In Bavaria, the first NPIs were permanently lifted in December 2021, after increasing COVID-19 vaccination rates and successful mitigation of the Omicron wave (13). In April 2022, most restrictions were lifted, including mask wearing obligation in schools, restaurants and shops, as well as regular testing obligation in schools (14). However, some restrictions like mask wearing obligation in public transport lasted until mid-December 2022, mask wearing obligation in medical care facilities even until April 2023 (15, 16).

As national and international findings lead to the hypothesis of a potential catch-up effect of several infectious diseases (11, 3), further investigation of post-NPI epidemiological trends is needed to get a better understanding of the short- and long-term impact of NPIs and their discontinuation. Therefore, the aim of this study was to assess how the incidence of infectious diseases other than COVID-19 changed since the discontinuation of COVID-19 NPIs in 2022 in Bavaria, Germany, compared to the pre-pandemic period.

## Methods

### Data source and inclusion criteria

We analyzed data collected for the surveillance of notifiable infectious diseases according to the German Infection Protection Act (Infektionsschutzgesetz, IfSG) and managed by the Bavarian Health and Food Safety Authority (Bayerisches Landesamt für Gesundheit und Lebensmittelsicherheit, LGL). According to §6 and §7 IfSG, clinicians and laboratories are obliged to report cases of certain infectious diseases and pathogens to local health authorities in less than 24 h (16). The reported cases are forwarded to the LGL within 24 h, which sends the notifications to the German national public health institute (Robert Koch-Institut, RKI) (16). We selected notified cases of influenza, chickenpox, norovirus gastroenteritis and rotavirus gastroenteritis reported between 1 January 2016 and 31 December 2022 in Bavaria. We chose to examine influenza, chickenpox, norovirus gastroenteritis, and rotavirus gastroenteritis due to their notably lower-than-expected case numbers during the pandemic, as determined by the counterfactual (4), and because they represent different modes of person-to-person transmission (droplets, airborne, fecal-oral) (17).

We included cases fulfilling case definitions by RKI. For influenza, a new reference definition was introduced in 2019. Before week 40–2019, only cases with a laboratory confirmation and information on clinical-epidemiological aspects fulfilled the definition. The new reference definition also includes cases with laboratory confirmation, for which no clinical diagnosis or epidemiological link was available. To take into account the change in reference definition for influenza after week 40–2019, we applied the new reference definition to the entire observation period. For chickenpox, rotavirus and norovirus gastroenteritis, there were no changes in requirements for notification (17).

### Statistical methods

To assess the impact of the COVID-19 pandemic on notified cases of the selected diseases in 2022, we performed time series analyses per disease considering weekly aggregated case numbers. We adopted the methodology by van de Berg et al. (4), extended by 2022. For each disease, we performed two types of analysis:

First, we forecasted weekly cases for 2020, 2021 and 2022 based on weekly case numbers in 2016–2019 for each disease (‘predictive model’). The purpose of this model was to simulate a counterfactual, to assess changes in case numbers in 2020, 2021 and 2022.

Specifically, we fitted a negative binomial regression model for each selected disease based on aggregated weekly case numbers from 2016 to 2019, including terms for trend and seasonality, model (a). These models were adopted from van de Berg et al. (4). Seasonal patterns were defined based on literature (17), supported by the assessment of disease-specific periodograms for weekly case numbers in 2016–2019 (18). Seasonality was accounted for in the negative binomial regression models by incorporating corresponding sine and cosine terms (4).

(a)
$$\begin{array}{c} \log\left(E\left(\mathrm{coun}t_{\mathrm{week}}\right)\right)=\alpha +\beta_{1}\times \mathrm{week}+\beta_{2}\sin\left(\frac{\mathrm{week}\times 2\pi \;}{52}\right)\\ +\beta_{3}\;\cos\left(\frac{\mathrm{week}\times 2\pi \;}{52}\right) \end{array}$$

For model selection, we considered three types of generalized regression models commonly used for count data: negative binomial regression, Poisson regression and quasi-Poisson regression (19). The relative goodness-of-fit was compared using the Akaike Information Criterion (AIC). Model fit was further assessed through residual analysis, which included a check for constant variance over time, autocorrelation (Ljung–Box test) and normal distribution (Shapiro–Wilk test).

We fitted predictive negative binomial regression models based on 2016–2019 data, and used the respective fitted models to determine expected weekly case numbers for 2020, 2021 and 2022, including respective 95% prediction intervals. We compared predicted with observed case numbers, focusing on weekly case numbers in 2022. We determined the number of weeks in which the observed case numbers were above or below the estimate or the 95% prediction intervals.

Although 2 March 2020 was marked as the start of the first COVID-19 wave in Germany (20), the cut-off date for the prediction period was marked 1 January 2020. We excluded reporting week 53–2020 from the analysis to obtain a consistent number of weeks for each year.

Second, we conducted an interrupted time series analysis to quantify the overall effect of the pandemic period on the incidence of the selected diseases compared to the pre-pandemic phase, focusing particularly on the period following the discontinuation of NPIs in 2022. For this purpose, we fitted negative binomial regression models including terms for trend and seasonality based on the full observation period (2016–2022) for each disease, model (b). We assessed the impact of different pandemic phases (relative change in case numbers for each pandemic year) by including an explanatory factor variable distinguishing the following periods in the model: 0 = Pre-pandemic (1 January 2016–1 March 2020); 1 = Pandemic Year 1 (2 March 2020–31 December 2020); 2 = Pandemic Year 2 (1 January 2021–31 December 2021); 3 = Pandemic Year 3 (1 January 2022–31 December 2022). Using disease specific periodograms, we assessed periodicity for the whole observation period (2016–2022), to ensure consistency with seasonal patterns identified in model (a). We derived disease-specific incidence rate ratios (IRR), which represent the ratio of the incidence rate during the pandemic period to the incidence rate in the pre-pandemic period, by exponentiating the coefficients (β_4_) from the regression models.

(b)
$$\begin{array}{c} \log\left(E\left(\mathrm{coun}t_{\mathrm{week}}\right)\right)=\alpha +\beta_{1}\times \mathrm{week}+\beta_{2}\sin\left(\frac{\mathrm{week}\times 2\pi \;}{52}\right)\\ +\beta_{3}\;\cos\left(\frac{\mathrm{week}\times 2\pi \;}{52}\right)+\beta_{4}\times \mathrm{period} \end{array}$$

Model choice and goodness-of-fit assessment were conducted as described for model (a). To improve model fit for influenza, additional variables were considered, including a binary variable for changes in case definitions and a binary variable to correct for the unusually severe influenza season 2017/2018. Additionally, a larger observation period for the pre-pandemic phase (2010–2019) was assessed. We evaluated model fit based on the AIC. However, the additional variables and longer observation period did not improve model fit and were therefore not included in the final model.

The significance level for hypothesis testing was set to 5% for all analyses. We performed all analyses with R (Version 4.3.1), using the packages tidyverse, MASS and trending (21).

## Results

### Change of weekly case numbers during the pandemic

In total, 367,356 cases were notified between 2016 and 2022. Excluding 17 cases from week 53–2020, we included 367,339 cases in the time series analyses (influenza *n* = 274,290, chickenpox *n* = 26,582, norovirus gastroenteritis *n* = 49,516, rotavirus gastroenteritis *n* = 16,951).

In the first two pandemic years, 2020 and notably 2021, the number of cases substantially decreased for all four selected diseases (Table 1). The decrease was most pronounced for influenza, with 152 notified cases in 2021 (pre-pandemic yearly average: *n* = 51,049). In 2022, case numbers increased again for all diseases to varying extents. The increase was highest for influenza (2021: *n* = 152; 2022: *n* = 55,251).

**Table 1:** Cases of selected notifiable infectious diseases reported in 2016–2022 (*n* = 367,339), in Bavaria, Germany, by period (Pre-pandemic = Week 1 of 2016 to Week 9 of 2020; Pandemic Year 1 = Weeks 10 to 52 of 2020; Pandemic Year 2 = Weeks 1 to 52 of 2021; Pandemic Year 3 = Weeks 1 to 52 of 2022) and respective incidence rate ratios (IRR) per period based on the interrupted time series models.

**Disease and period**	**Cases, *n***	**IRR (95% CI)**	***p*-value**
**Influenza**			
Pre-pandemic (yearly average)	204,196 (51,049)		
Pandemic Year 1	14,691	0.97 (0.52–1.83)	0.920
Pandemic Year 2	152	0.03 (0.01–0.06)	<0.001
Pandemic Year 3	55,251	3.47 (1.49–7.94)	0.001
**Chickenpox**			
Pre-pandemic (yearly average)	21,435 (5,359)		
Pandemic Year 1	1,476	0.40 (0.34–0.47)	<0.001
Pandemic Year 2	1,356	0.30 (0.25–0.37)	<0.001
Pandemic Year 3	2,315	0.52 (0.41–0.65)	<0.001
**Norovirus gastroenteritis**			
Pre-pandemic (yearly average)	39,080 (9,770)		
Pandemic Year 1	1,075	0.15 (0.12–0.19)	<0.001
Pandemic Year 2	3,574	0.40 (0.30–0.54)	<0.001
Pandemic Year 3	5,787	0.59 (0.42–0.82)	<0.001
**Rotavirus gastroenteritis**			
Pre-pandemic (yearly average)	12,951 (3,238)		
Pandemic Year 1	492	0.32 (0.24–0.42)	<0.001
Pandemic Year 2	671	0.34 (0.26–0.44)	<0.001
Pandemic Year 3	2,837	1.36 (0.95–1.93)	0.08

CI: confidence interval, IRR: incidence rate ratio.

Based on our interrupted time series models, we identified a higher incidence rate compared to the pre-pandemic period for influenza (IRR = 3.47, 95%CI: 1.49–7.94) and for rotavirus gastroenteritis (IRR = 1.36, 95%CI: 0.95–1.93) in 2022. The incidence rate in 2022 remained below pre-pandemic levels for chickenpox (IRR = 0.52, 95% CI: 0.41–0.65) and norovirus gastroenteritis (IRR = 0.59, 95% CI: 0.42–0.82) (Table 1).

**Figure 1:**
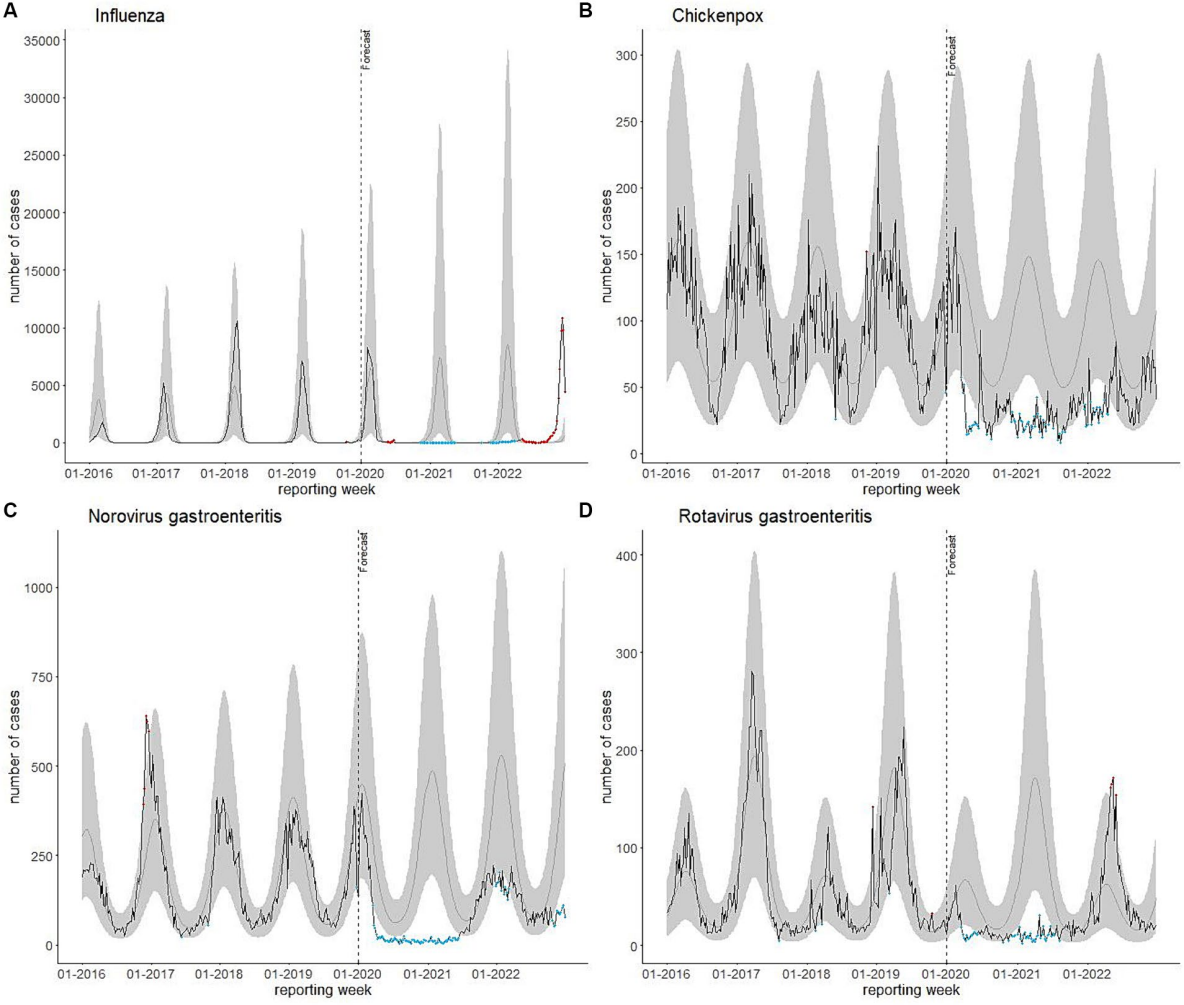
**(A)** Influenza, **(B)** chickenpox, **(C)** norovirus gastroenteritis, and **(D)** rotavirus gastroenteritis cases reported per reporting week in Bavaria, Germany, 2016–2022 (*n* = 367, 339) plotted against predicted weekly case numbers based on notifications of 2016–2019. Black line: reported cases; grey line: predicted cases; shaded areas: 95% prediction intervals. Blue dots: weeks in which case numbers were below the 95% prediction interval; orange dots: weeks in which case numbers were above the 95% prediction interval.

Results from the model that predicted case numbers for 2020, 2021 and 2022 based on pre-pandemic years underline these findings (Figure 1; Table 2). For influenza, in 63.5% of the weeks in 2022, case numbers were above the 95% prediction interval. For chickenpox and norovirus gastroenteritis, case numbers in most weeks were either below the estimate (chickenpox: 92.3%; norovirus gastroenteritis: 88.5%) or even below the 95% prediction interval (chickenpox: 26.9%; norovirus gastroenteritis: 36.5%). For rotavirus gastroenteritis, in 67.3% of the weeks, case numbers were above the estimate or above the 95% prediction interval (7.7%).

**Table 2:** Percentage of weeks in 2020, 2021 and 2022 with case numbers within the calculated 95% prediction interval (below and above estimate) and outside the 95% prediction interval [below lower and above upper prediction interval (PI)].

	Year	Weeks below PI	Weeks below point estimate	Weeks above point estimate	Weeks above PI
Influenza	2020	11.5%	65.4%	26.9%	11.5%
	2021	51.9%	98.1%	1.9%	0.0%
	2022	26.9%	32.7%	67.3%	63.5%
Chickenpox	2020	38.5%	90.4%	7.7%	0.0%
	2021	61.5%	100.0%	0.0%	0.0%
	2022	26.9%	92.3%	7.7%	0.0%
Norovirus gastroenteritis	2020	78.8%	98.1%	7.7%	0.0%
	2021	48.1%	86.5%	0.0%	0.0%
	2022	36.5%	88.5%	7.7%	0.0%
Rotavirus gastroenteritis	2020	34.6%	88.5%	5.8%	0.0%
	2021	55.8%	80.8%	17.3%	0.0%
	2022	0.0%	30.8%	67.3%	7.7%

### Changes in seasonality

Seasonality can also be assessed in Figure 1. While the pre-pandemic seasonal increases in case numbers for the diseases selected were absent or lower during the pandemic, in 2022 pre-pandemic seasonal patterns mostly reoccurred, except for influenza. The influenza season 2022/2023 was characterized by an unusual early wave starting around week 39–2022. Our model estimated influenza case numbers in 2022 to increase from week 47 and to continuously increase until the end of the year. However, notified case numbers peaked in week 50 with 10,822 reported cases, followed by a decline to 4,401 in week 52. The rotavirus gastroenteritis seasonal increase of case numbers reoccurred between April and June 2022 similar to the pre-pandemic seasonality, though with relatively high case numbers. For chickenpox and norovirus gastroenteritis, the pre-pandemic seasonal pattern characterized by high numbers during winter months returned in the winter season 2021/2022. However, case numbers remained below pre-pandemic levels. For chickenpox, observed weekly case numbers returned to be within the prediction interval from week 172,022. Contrary to the prediction, there was no increase in case numbers of norovirus gastroenteritis in late 2022.

## Discussion

This study aimed to determine how the incidence of selected infectious diseases other than COVID-19 changed since the discontinuation of COVID-19 NPIs in 2022 as compared to the pre-pandemic period. We hypothesized to see a catch-up effect of infections after a significant decrease in cases in a numbers of infectious diseases Bavaria in 2020 and 2021 (4). The lack of immune stimulation potentially led to increased community immunity in 2022 including the risk of large outbreaks (22). Our results suggest complex epidemiological dynamics for each infectious disease. While the hypothesis of a catch-up effect was not confirmed for all selected diseases, our analyses affirm a notable rebound of some of the considered infectious diseases. It is necessary to discuss the disease-specific findings individually and put them into context and disease-specific aspects of transmission.

### Influenza

After a low influenza activity during the first 2 years of the COVID-19 pandemic, in 2022 case numbers returned to high pre-pandemic levels, as indicated by a significantly higher IRR in 2022. While at the beginning of 2022, case numbers remained low – presumably due to strict NPIs still in place – weekly case numbers were higher than expected for most of the year as estimated in our model. An unusually early start in October and high case numbers peaking in the last weeks of the year characterized the influenza season 2022/2023. Pre-pandemic influenza waves usually started in January and peaked around February. Our findings are consistent with other national and international findings based on routine surveillance data (23), sentinel specimen data (10, 24) and on paediatric hospital admission rates (25).

The increase in influenza case notifications may partially be a result of increased testing and health care seeking behavior. In 2022, persons experiencing respiratory symptoms may have been increasingly tested due to COVID-19 testing requirements, resulting in a higher number of other respiratory infections, including influenza, being detected as differential diagnosis (24).

However, there are several factors that would explain a real increase in influenza transmission. Less exposure to the influenza virus in the first pandemic years may have led to decreased community immunity and therefore a higher proportion of susceptible individuals (3, 26). Vaccination coverage among people aged 60 years and older in Bavaria slightly decreased from 36.6% in 2020/2021 to 32.2% in 2021/2022, possibly leading to less vaccine-induced immunity in the population (27, 28). However, influenza vaccine effectiveness must also be taken into account, which can vary from season to season, depending on factors such as the degree of antigenic match between the influenza strains in the vaccine and circulating strains (29).

The unusually early start of the influenza wave could possibly be due to an interference or viral block between COVID-19 and influenza (30). There may have been an antagonistic competition between COVID-19 and influenza, potentially leading to a lower risk of co-infections and shifts in seasonality (26, 30). This phenomenon was seen in many other respiratory viral coinfections (30). In general, influenza waves are difficult to predict due to various contributing seasonal characteristics, like subtypes, contact behavior, and vaccine coverage/effectiveness, which explains the heterogeneous and dynamic nature of influenza circulation, also reflected by our data (31).

### Chickenpox

Case numbers for chickenpox increased in 2022, but the incidence overall remained significantly lower than in pre-pandemic years. A possible resurgence of chickenpox after the lifting of NPIs was not confirmed by our analysis. Our findings are consistent with surveillance data by RKI on a national level, as the total number of notified cases for 2022 were less than half compared to pre-pandemic years (23). In contrast to these findings, a Chinese study found an increase of chickenpox cases in the late pandemic stages (32).

The following factors could explain the continuously low transmission of chickenpox in 2022: It is likely that NPIs still affected chickenpox case numbers in 2022. During the estimated chickenpox wave in the first months of 2022, various COVID-19 related restrictions were still in place, such as mandatory mask wearing and fixed groups in childcare facilities (15). Only during the following wave, starting in late 2022, most of the NPIs were discontinued. However, the full extent of the wave is not covered by our data. Furthermore, increasing vaccine rates during the pandemic may have led to fewer chickenpox transmissions. School entry medical data reports an increase of varicella vaccine rates from 76.7% in 2017/2018 to 81.4% in 2020/2021 in Bavaria (33).

The decrease in case numbers in 2022 is likely not attributable to reduced healthcare-seeking behavior. According to the Central Institute for Statutory Health Insurance Physicians in Germany, utilization of services by contracted physicians returned to pre-pandemic levels or even exceeded them in the first half of 2022 (34).

### Norovirus gastroenteritis

Similar to chickenpox, norovirus gastroenteritis activity in 2022 was below the pre-pandemic average. Pre-pandemic seasonal patterns with higher case numbers during the winter months reoccurred, but at a lower level. The norovirus gastroenteritis wave peaked in week 50–2021 with 223 cases observed.

These findings are in line with national routine surveillance data, showing an increase in case numbers in 2022 compared to 2021. However, the reported numbers were still only half of those observed during the pre-pandemic years (23). In contrast to our findings, studies from England and China found a marked increase in norovirus gastroenteritis outbreaks after the ease of NPIs (8, 35).

Strict COVID-19 measures during the 2021/2022 norovirus gastroenteritis wave may have led to a milder wave with fewer outbreaks. Nevertheless, it is surprising that norovirus gastroenteritis case numbers remained low at the end of the year 2022 as well, although most NPIs were lifted at that time. This could be due to better hygiene measures following the pandemic, particularly during the winter months (32). Similar to chickenpox, decreased health care seeking may not have played a role in 2022 anymore, as outpatient care data indicates (34).

### Rotavirus gastroenteritis

In contrast to norovirus gastroenteritis, case numbers for rotavirus gastroenteritis rebounded back to pre-pandemic levels, even exceeding the estimated values based on the pre-pandemic period during the weeks with highest peaks. National routine surveillance data show a similarly notable increase of case numbers in 2022 (23).

The surge in rotavirus gastroenteritis cases in 2022 may represent a rebound of infections. Rotavirus gastroenteritis is particularly incident among small children younger than 5 years (17). Due to the low transmission of rotavirus while NPIs were in place, many children likely remained unexposed to the virus. The increase in the number of susceptible individuals may explain the observed rise of cases in 2022. It underlines the importance of rotavirus vaccination coverage for infants (36). Our results further raise the hypothesis of a shift in the periodic pattern. In pre-pandemic times, a biennial pattern has been observed in our data, which is common in countries with moderate to high vaccination coverage (37). The possible increase in the susceptible population may have led to the higher peak in cases occurring in 2022 instead of 2021 as forecasted, possibly shifting also the future biennial pattern.

In summary, our findings only partly confirm a catch-up effect of notifications of infectious diseases after lifting NPIs. We observed an increase in case number of rotavirus gastroenteritis, though not significant, at a time where most NPIs were lifted. Similarly, there was a significant increase in influenza case numbers outside the usual season. Norovirus gastroenteritis and chickenpox case numbers remained low in 2022. Possibly, this is partly due to some NPIs still being in place in early 2022 when the peaks in cases were expected.

### Strengths and limitations

A strength of our analysis of routine surveillance data is that it allows for a long-term assessment of infectious disease case numbers during and after the COVID-19 pandemic and comparison with routine reporting data from other sources. Such analyses may inform future preparedness and response to infectious disease outbreaks.

Our study, however, has some limitations. Defining four time periods for assessing the effects of the pandemic in our regression modeling represents a simplified approach, which do not allow to detect incidence changes within these periods. This simplification also leads to difficulties in interpreting the impact of lifting NPIs, as in early 2022 a number of NPIs were still in place. Our model, however, is suitable to identify general trends in infectious disease dynamics. In addition, to ensure comparability to the previous results by van de Berg et al. (4), we chose the pragmatic approach of four different pandemic periods. We would like to reiterate that causality between NPIs and the incidence of notifiable diseases cannot be inferred on the sole basis of the considered model and data.

Furthermore, our data only includes notifications until and including week 52–2022. For diseases characterized by high case numbers during this time, only a part of the wave is covered by our data. To better understand infectious disease dynamics, particularly for chickenpox and norovirus gastroenteritis, the analyses need to be extended to include further data.

Particularly for influenza, regression modeling was challenging partly due to the relatively high variation of influenza waves in the pre-pandemic years (31). Modeling diagnostics indicated a suboptimal fit of the regression model, suggesting heterogeneity not captured by our approach. Nevertheless, we are confident that the regression model reproduces the overall course of case numbers reasonably well for drawing conclusions.

When interpreting the results presented in this study, it is critical to note that there may be multiple explanatory factors for our observations, which are not included in the surveillance data. Parameters like the effectiveness of vaccination/immunization status, climate influences, the role of changing contact patterns or health awareness/hygiene could not be taken into consideration.

Furthermore, surveillance data do not represent the real incidence of a disease in a population. Disease notifications depend on health care seeking, the diagnosis by physicians and laboratories, they are likely characterized by an overrepresentation of severe cases and an underestimation of total case numbers. We cannot determine to what extent the changes in case numbers are explained by a true change in transmissions or by changes in notifications of infectious diseases.

## Conclusion

Our study adds to the understanding of how selected infectious diseases reoccurred after COVID-19 NPIs were gradually lifted. The results indicate distinct patterns per selected disease, only partly confirming the hypothesis of a catch-up effect, i.e., a higher incidence in 2022 compared to pre-pandemic times. Further monitoring of epidemiological trends will be needed to identify the overall infectious disease dynamics after the long-term discontinuation of COVID-19 NPIs. This includes the monitoring of other data sources, like hospitalization data and primary care data, as these provide important information on potential patient surges and other relevant aspects for public health. To facilitate this, existing syndromic surveillance programmes may be expanded to enable triangulation with routine surveillance data. Further investigation of the independent and synergistic effects of specific NPIs on infectious disease activity and the role of other contributors (e.g., vaccine coverage) and biological mechanisms (e.g., viral interference) is needed. Ideally, federal or national health registries may be established including, e.g., vaccination records to allow analysis adjusted for other health factors.

Future pandemic preparedness plans should consider the potential unintended effects of NPIs. While NPIs have effectively reduced the burden of COVID-19, they may have also led to catch-up phenomena and broader impacts on physical and mental health. These plans should emphasize maintaining high hygiene standards beyond the pandemic, especially in high-risk settings for outbreaks. Additionally, they should incorporate measures to ensure continued, universal access to and use of healthcare services, including vaccination, throughout pandemics.

## Data availability statement

The data analyzed in this study is subject to the following licenses/restrictions: detailed data are confidential and protected by German law (IfSG) and are available from the corresponding author upon reasonable request. Aggregated data from a limited version of the German surveillance system database can be retrieved via SurvStat@RKI 2.0; https://survstat.rki.de/. Requests to access datasets should be directed to judith.hausmann@lgl.bayern.de.

## Ethics statement

Ethical approval was not required for the study involving humans in accordance with the local legislation and institutional requirements. Written informed consent to participate in this study was not required from the participants or the participants’ legal guardians/next of kin in accordance with the national legislation and the institutional requirements.

## Author contributions

JH: Writing – original draft, Writing – review & editing, Conceptualization, Methodology, Formal analysis, Visualization. AD: Writing – review & editing, Conceptualization, Supervision, Methodology. KK: Writing – review & editing, Conceptualization, Supervision. SB: Conceptualization, Methodology, Writing – review & editing, Formal analysis, Supervision.
