# Corrigendum: Lifting COVID-19-associated non-pharmaceutical interventions: potential impact on notifications of infectious diseases transmitted from person to person in 2022 in Bavaria, Germany

**DOI:** 10.3389/fpubh.2024.1544751

**Published:** 2025-01-07

**Authors:** Judith Hausmann, Achim Dörre, Katharina Katz, Sarah van de Berg

**Affiliations:** ^1^Institute for Medical Information Processing, Biometry, and Epidemiology - IBE, LMU Munich, Munich, Germany; ^2^Pettenkofer School of Public Health, Munich, Germany; ^3^Bavarian Health and Food Safety Authority (LGL), Munich, Germany; ^4^Department of Infectious Disease Epidemiology, Postgraduate Training for Applied Epidemiology (PAE), Robert Koch Institute, Berlin, Germany

**Keywords:** infectious diseases, surveillance, pandemic, non-pharmaceutical interventions, influenza, chickenpox, norovirus gastroenteritis, rotavirus gastroenteritis

In the published article, the reference (1) in “These dynamics were observed for respiratory syncytial virus (RSV) (7), rhinovirus infections (1), hand, foot, and mouth disease (2), norovirus gastroenteritis (8), and influenza (9). As children were predominantly affected (1, 7, 8), concerns arose that this phenomenon would appear in other infectious diseases, like rotavirus gastroenteritis or chickenpox as well (10)” was incorrectly specified as “Ullrich, A, Schranz, M, Rexroth, U, Hamouda, O, Schaade, L, Diercke, M, et al. Impact of the COVID-19 pandemic and associated non-pharmaceutical interventions on other notifiable infectious diseases in Germany: an analysis of national surveillance data during week 1-2016 - week 32-2020. *Lancet Reg Health Eur*. (2021) 6:100103. doi: 10.1016/j.lanepe.2021.100103.” In the above mentioned sentences it should be replaced by “Maison, N, Peck, A, Illi, S, Meyer-Buehn, M, von Mutius, E, Hübner, J, et al. The rising of old foes: impact of lockdown periods on “non-SARS-CoV-2” viral respiratory and gastrointestinal infections. *Infection*. (2022) 50:519–24. doi: 10.1007/s15010-022-01756-4.”

The authors apologize for this error and state that this does not change the scientific conclusions of the article in any way. The original article has been updated.

